# Yellow Passion Fruit Seed Meal (*Passiflora edulis* f. *flavicarpa*) as a Feed Ingredient for Fattening Steers: Effects on Intake, Weight Gain, *In Vitro* Fermentation and Feed Cost

**DOI:** 10.3390/ani16162513

**Published:** 2026-08-12

**Authors:** Marlon Carlosama-Yépez, Adrián Vélez, Jennifer Cuenca, Diego Melo-Durán, Katherine Moreira, Francisco Gutiérrez, Andrea Cortes

**Affiliations:** 1 Dirección General de Postgrados, Universidad UTE, Quito 170527, Ecuador; adrianm.velez@ute.edu.ec (A.V.); jennifer.cuenca@ute.edu.ec (J.C.); diego.melo@ute.edu.ec (D.M.-D.); fgutierrez@uce.edu.ec (F.G.); andrea.cortesv@ute.edu.ec (A.C.); 2 Faculty of Veterinary Medicine and Agronomy, Universidad UTE, Quito 170147, Ecuador; 3 Faculty of Veterinary Sciences, Universidad Técnica de Manabí, Portoviejo 130103, Ecuador; yessenia.moreira@utm.edu.ec; 4 Facultad de Ciencias Agrícolas, Universidad Central del Ecuador, Quito 170521, Ecuador

**Keywords:** passion fruit seed meal, beef cattle, growth performance, *in vitro* fermentation, feed cost

## Abstract

The use of fruit by-products in animal feed can reduce costs and competition between human and animal feed. This study evaluated the effect of dietary inclusion of yellow passion fruit (*Passiflora edulis* f. *flavicarpa*) seed meal (PSM) on growth performance, *in vitro* fermentation, and feed costs in beef steers. A total of 36 steers (*Bos indicus*; initial BW 416 ± 30 kg) were randomly assigned to three treatments: a control diet (CON) and diets containing 5 or 10% PSM for 45 days. Feed intake and average daily gain (ADG) were measured. An *in vitro* fermentation assay evaluated gas production and pH changes. Steers fed with 5% and 10% PSM showed numerically higher group-level fresh matter intake (1.10% and 9.34%, respectively), and significantly greater ADG over the entire trial (*p* = 0.00015) compared to CON. In addition, diets containing 10% PSM produced the highest cumulative gas (17.00 ± 1.00 mL vs. 10.00 ± 1.00 mL for CON and 10.33 ± 2.52 mL for 5% PSM; *p* = 0.003), indicating high fermentability. Feed cost was reduced by 57% and 46% when using diets containing 5% and 10% PSM, respectively. In conclusion, this study reveals that dietary inclusion of up to 10% PSM improves feed intake and growth performance in steers while reducing feed costs, supporting its value as a sustainable feed ingredient for beef cattle.

## Simple Summary

This study investigated whether adding a meal made from yellow passion fruit seeds, a by-product of juice production in Ecuador, to beef cattle diets could be beneficial for growth performance and feed cost. We fed young steers with either a standard diet or diets containing 5 or 10% passion fruit seed meal for 45 days. We found that the steers preferred the taste of the feed containing the seed meal, leading them to eat more. This resulted in a significantly higher daily weight gain compared to those on the standard diet. Laboratory tests confirmed that the seed meal was fermentable in the rumen without causing harm. Most importantly, using this by-product reduced feed cost’s share of revenue to 46–57%, down from 83% in the control. This research shows that incorporating passion fruit seed meal into cattle feed is a promising strategy because not only lowers production costs for farmers and improves animal growth, but also adds value to an agricultural by-product, reducing competition between human food and animal feed.

## 1. Introduction

Ecuadorian beef cattle production relies extensively on grass feeding systems. However, during dry seasons, farmers depend on a combination of fermented feed (silage) and pelleted concentrates. In this context, production units face uncertainty due to fluctuating prices of the main grain components in rations, such as soy and corn. This fact has led farmers to formulate rations with less expensive ingredients such as plant by-products. Among these ingredients, yellow passion fruit seed meal (PSM) has emerged as a cost-effective option for beef production farms, especially in Esmeraldas, Santo Domingo de los Tsáchilas, and Manabí provinces, where the main production of Ecuadorian yellow passion fruit (*Passiflora edulis* f. *flavicarpa)* is located [1].

Production costs in cattle systems are linked to the level of intensification, with intensive systems requiring higher supplementation rates [2]. In the United States of America, feed costs alone account for 60–75% of operating expenses in beef steer production [3]. Similarly, in Argentina, feed is the central and most expensive component of the system, accounting for 65–75% of costs, depending largely on corn prices and diet composition [4]. In contrast, pastoral systems are less dependent on purchased feed. For instance, steer production on megathermic pastures in Mexico reported that feed costs accounted for only 27% of the total [5]. In the Ecuadorian context, the inclusion of locally available agro-industrial by-products, such as PSM, may be a strategic option for reducing feed costs.

In 2023, Ecuador produced 76,030 tons of fresh yellow passion fruit; of these, 16,728 tons were exported as juice or fresh fruit [1]. The industrial process to obtain passion fruit juice involves pulp extraction, seed separation, and pressing. One of the by-products is passion fruit seed meal (PSM). While the juice industry yields substantial quantities of PSM (approximately 15–20% of processed fruit weight) [6], the animal feed sector is underutilising this by-product, consistent with broader patterns of limited use of fruit processing by-products in livestock diets [7]. This is attributed to the unknown optimal proportions of PSM in cattle rations, its effects in the rumen, and a lack of knowledge of its nutritional value.

**Figure 1:**
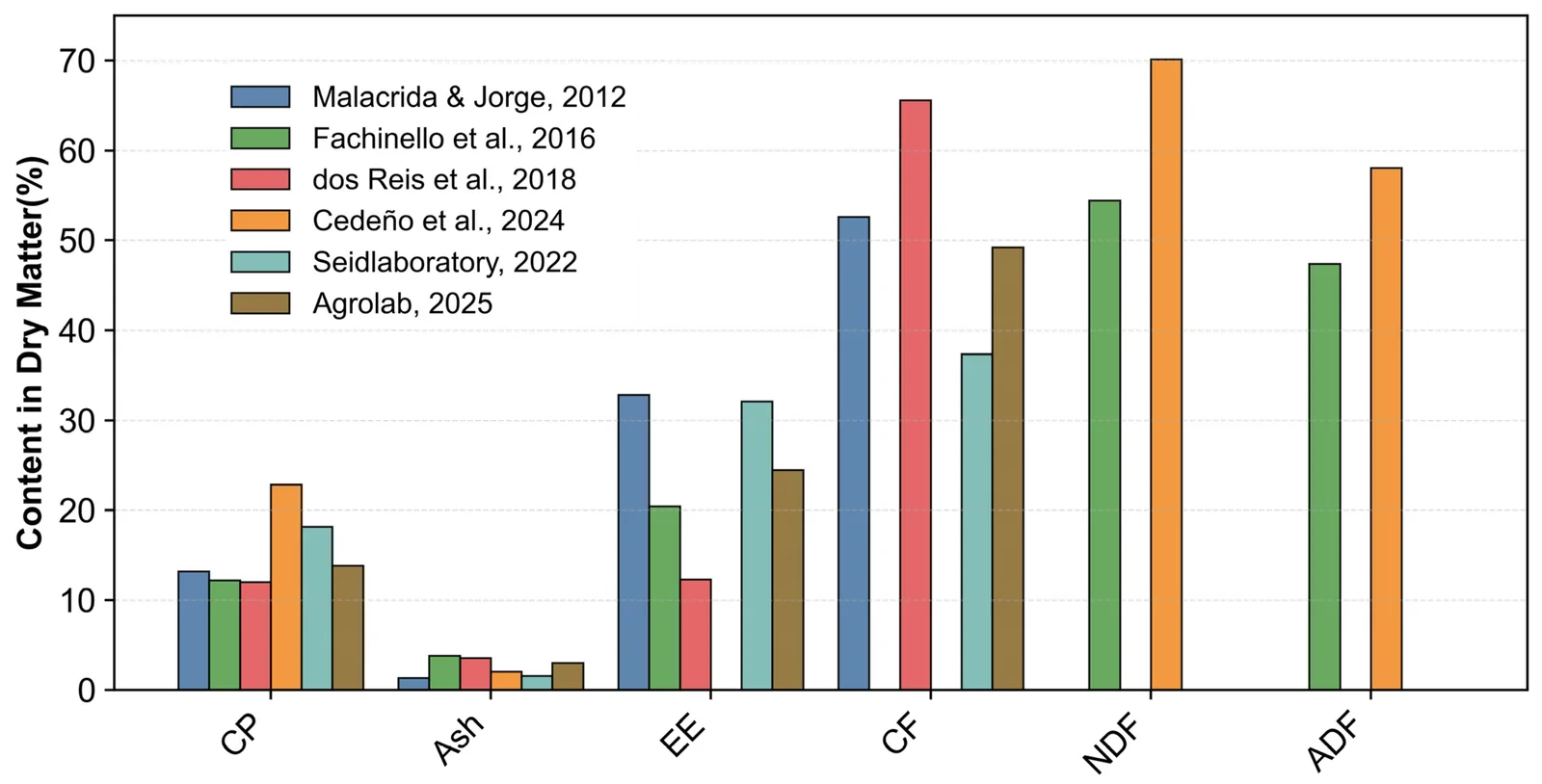
Comparative nutrient composition of yellow passion fruit seed meal (PSM) from various sources, presented on a dry matter (DM) basis. To align with standard animal nutrition practices, the protein conversion factor from dos Reis et al. [8] was adjusted from 5.5 to 6.25 for crude protein (CP) calculations. The values reported by Malacrida and Jorge [9] for the sum of Fiber and carbohydrates are presented here as Crude Fiber (CF). Analyses from Seidlaboratory (Figure S1) and Agrolab Laboratories (Figure S2) were conducted on samples provided by the passion fruit seed meal supplier in 2022 and 2025, respectively. EE, Ether Extract; NDF, Neutral Detergent Fibre; ADF, Acid Detergent Fibre [7,12].

Previous studies have analysed the chemical and nutritional components of yellow passion fruit seed (Figure 1). For instance, crude fibre content was consistently high across the analyses (51 ± 10%). This value is twice that of the media ether extract content and approximately 30% of the average protein content. Furthermore, the average mineral content was 2.57%, containing mainly potassium, phosphorus, and calcium, at 760, 310, and 30 mg/100 g dry matter (DM), respectively [7,8,9].

The observed variability among studies was anticipated, as the nutritional composition of plant-based ingredients is inherently influenced by factors such as climatic conditions, vegetative stage, ripeness, and processing methods [10]. Notably, fibre content is generally high. Considering the fibrous composition of PSM and the high capacity of ruminants to ferment these materials, the use of this by-product offers considerable potential not only for local beef productivity but also for reducing competition between humans and livestock for human-edible feed ingredients [11].

Regarding fatty acid composition, yellow passion fruit seed oil contains seven times more unsaturated fatty acids (UFA) than saturated fatty acids (SFA). For instance, Linoleic acid (C18:2 n-6) was the most abundant (73.14%) of the total UFAs, followed by oleic acid (C18:1 n-9) (13.83%). Therefore, the use of yellow passion derivatives may improve diet quality [9]. Apart from its nutrient value, yellow passion fruit seeds contain bioactive compounds such as phenolics (346.69 ± 6.58 ug/100 g DM), flavonoids such as kaempferol (375.32 ± 13.50 ug/100 g DM) and anthocyanins as malvidin 3,5-Di (4598.70 ± 119.73 ug/100 g DM) [8]. Some of these compounds may positively affect the ruminal microbiota and, eventually, performance. In fact, tannins (phenolics) and flavonoids have been shown to reduce rumen protein degradation, alter volatile fatty acid profiles and suppress methanogenic archaea in a dose-dependent manner [13,14,15,16].

Anecdotal reports from farmers in Santo Domingo de los Tsáchilas, who have empirically used PSM in cattle rations with observed improvements in body weight, are supported by Alves et al. [17], who observed that steers fed fresh passion fruit by-products exhibited greater weight gain and feed intake than those fed sorghum silage. In fact, the group evaluated fresh passion fruit peel and seeds as a complete forage replacement for Nelore steers, reporting an ADG of 1.30 kg/day versus body mass loss (−0.08 kg/day) in sorghum silage-fed animals. However, that study used unprocessed by-product as a forage substitute rather than processed PSM as a partial concentrate replacement, and included no economic analysis. These differences in product form, inclusion strategy, and economic evaluation define the gap that the present study addresses. Specifically, we evaluated growth performance, *in vitro* fermentation, and feed costs at 5 and 10% PSM inclusion levels.

## 2. Materials and Methods

### 2.1. Steer Experiment

#### Animals and Housing

**Figure 2:**
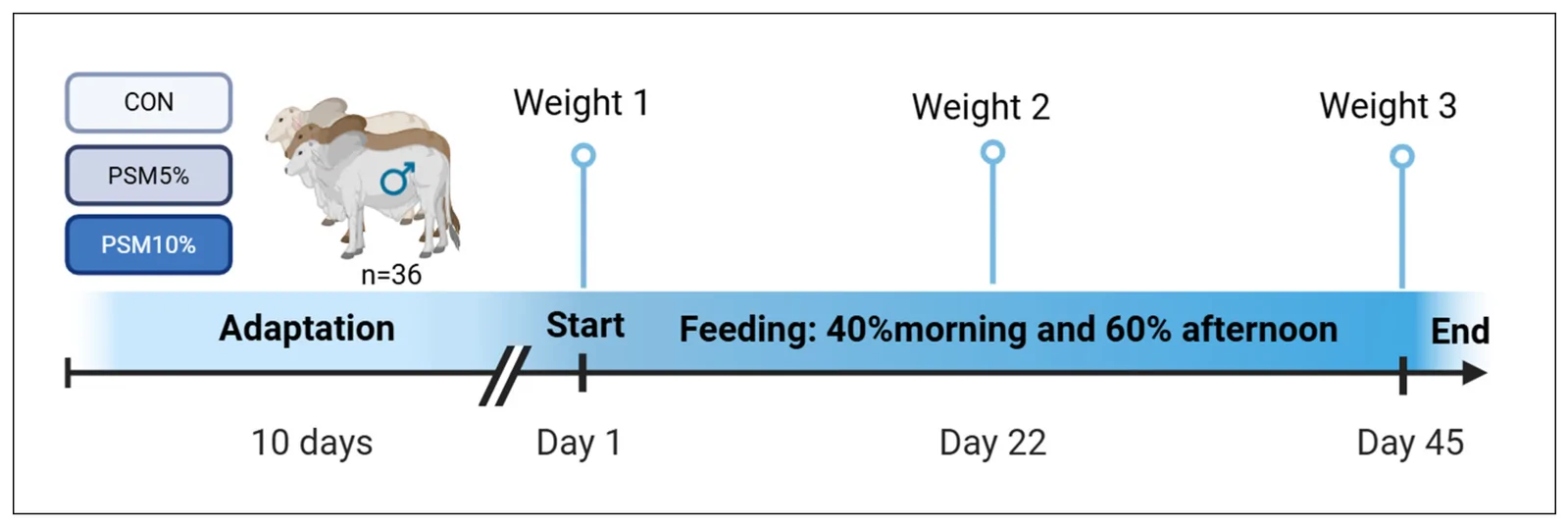
Experimental Procedure Stages. CON: Control diet; PSM5%: yellow passion fruit seed meal at 5%; PSM10%: yellow passion fruit seed meal at 10%.

A total of thirty-six (*n* = 36) crossbred steers (*Bos indicus* × *Bos taurus,* approximately 75% Brahman and 25% Holstein), ranging between 26 and 27 months old with an initial average body weight (BW) of 416 ± 30 kg, were randomly allocated into three treatment groups, with each treatment assigned to one pen (12 animals per pen) (Figure 2). The study followed a completely randomised design (CRD) with three treatments: a control diet (CON), a 5% PSM diet, and a 10% PSM diet. For feed intake, the pen (*n* = 1 per treatment) was the experimental unit; for average daily gain (ADG), the individual steer (*n* = 12 per treatment) was the experimental unit. The trial was conducted at the “El Progreso” beef cattle farm, located in La Concordia canton, Santo Domingo de los Tsáchilas province, Ecuador. This tropical region is located at an altitude of 217 m above sea level. It is characterised by a relative humidity exceeding 85%, mean temperatures ranging from 25 to 27 °C, and an annual precipitation of approximately 3000 mm.

Fresh matter intake was calculated as the difference between the fresh feed offered and refusals for each treatment group. Additionally, body weight was individually recorded using a fixed electronic scale(Model VNS203; VNS, Beijing, China) installed in the handling pen. The average initial weight was the same across treatment groups (*p* = 0.98).

### 2.2. Ethics and Welfare

The animal study protocol was approved by the Ethics Committee for Animal Research of Universidad UTE, with approval number CEIA-UTE-2025-005 granted on 12 May 2025. All procedures were conducted in strict compliance with national regulations for animal research as stipulated by the Agency for Regulation and Phytosanitary and Zoosanitary Control of Ecuador (AGROCALIDAD), in accordance with Resolution 227 [18]. The 36 steers were equally distributed among three 60 m^2^ group pens (*n* = 12 per treatment), providing 5 m^2^ per animal in accordance with the National Academies of Sciences, Engineering, and Medicine [19] recommendations, thereby ensuring adequate space for welfare. Throughout the experiment, all animals had ad libitum access to water and were under permanent veterinary supervision, enabling daily monitoring of their general health to ensure the absence of clinical signs, behavioural alterations, or disorders related to the dietary transition.

### 2.3. Diets and Analyses

Experimental diets were formulated on a dry matter (DM) basis to meet the nutritional requirements for growing steers [19]. Feed was offered at a fixed 10 kg DM steer^−1^ day^−1^ based on initial BW (2.5%). As BW increased from the initial 416 kg to approximately 493 kg by Day 45, the effective offer rate declined to about 2.0% of BW. Refusals were recorded daily and remained below 5% throughout the trial, indicating that the offer rate was sufficient to maintain ad libitum intake.

Calculated values were used for diet formulation. Analysed values (from wet chemistry) showed variability, likely due to heterogeneity in ingredient sampling. For treatment comparisons, we used the calculated values for crude protein (CP) and metabolizable energy (ME), as these were the intended formulation targets. The analysed values are reported in Table 1. Nutritional composition values were calculated from chemical analyses of individual ingredients, using a weighted average based on their proportional inclusion in each diet.

The proximate composition (Weende Analysis) was determined according to conventional methods described by the Association of Official Analytical Chemists [20]. Moisture analyses were performed by gravimetry. The principle of this method is based on the loss of mass (water) from the sample, whereby the sample is dried in a container at 135 °C for 2 h. The mass difference corresponds to the amount of water that evaporated during heating. DM was calculated as the difference between the initial and final weights of the sample after 24 h of drying. Ash percentage was determined by heating the samples at 600 °C for 6 h using a muffle furnace (Thermo Scientific, Waltham, MA, USA). Crude fibre content was determined gravimetrically, following acid and alkaline extraction or digestion. Crude protein (N × 6.25) was determined using the Kjeldahl method with a Kjeldahl distiller (Pro-Nitro M, Selecta, Abrera, Spain), and crude fat was determined using the Soxhlet method with a fat extractor (Det-Gras N, Selecta, Abrera, Spain), respectively [20].

### 2.4. In Vitro Fermentation Experiment

Fermentability of increasing concentrations of PSM (CON, 5% PSM and 10% PSM) was assessed in an *in vitro* trial following the gas production method described by Theodorou et al. [21]. In brief, fresh rumen fluid, artificial saliva (McDougall buffer), and PSM-based feed were used. Each treatment was fermented in triplicate (*n* = 3), consistent with standard practice and sufficient to detect treatment effects [22,23]. A total of 12 syringes were incubated for 12 h at 39 °C. This experiment was carried out at Universidad Laica Eloy Alfaro in Manabí, and the bromatological analyses were performed at Universidad Técnica de Manabí in Ecuador.

#### 2.4.1. Preparation of the Artificial Saliva

The artificial saliva was prepared according to the formulation described by McDougall [24] and adapted for this experiment. Briefly, the solution was prepared using 29.4 g of sodium bicarbonate, 21 g of dibasic phosphate, 2.91 g of sodium chloride, 1.71 g of potassium chloride, 0.36 g of magnesium sulphate, and 3 mL of calcium chloride (4%). After homogenising the mixture, it was transferred to a 2000 mL flask. Distilled water was then added to bring the final volume to 2500 mL. The initial pH of the solution was between 8.0 and 8.5; therefore, carbon dioxide was bubbled through it to reduce the pH to 6.8–7.0. The artificial saliva solution was subsequently mixed with fresh rumen fluid at a 3:1 ratio [21].

#### 2.4.2. Rumen Fluid

Fresh rumen fluid was aseptically collected from an approximately 12-month-old crossbred Nelore steer (*Bos indicus*) within 30 min post-slaughter. The animal was processed at the Empresa Pública Mancomunada del Trópico Húmedo slaughterhouse (AGROCALIDAD register number 23-003) in Santo Domingo de los Tsáchilas. All procedures were conducted under conditions designed to preserve microbial viability [21]. Rumen fluid was immediately transferred to pre-warmed (39 °C), thermally insulated containers and transported to the laboratory.

#### 2.4.3. Gas Production and pH

**Figure 3:**
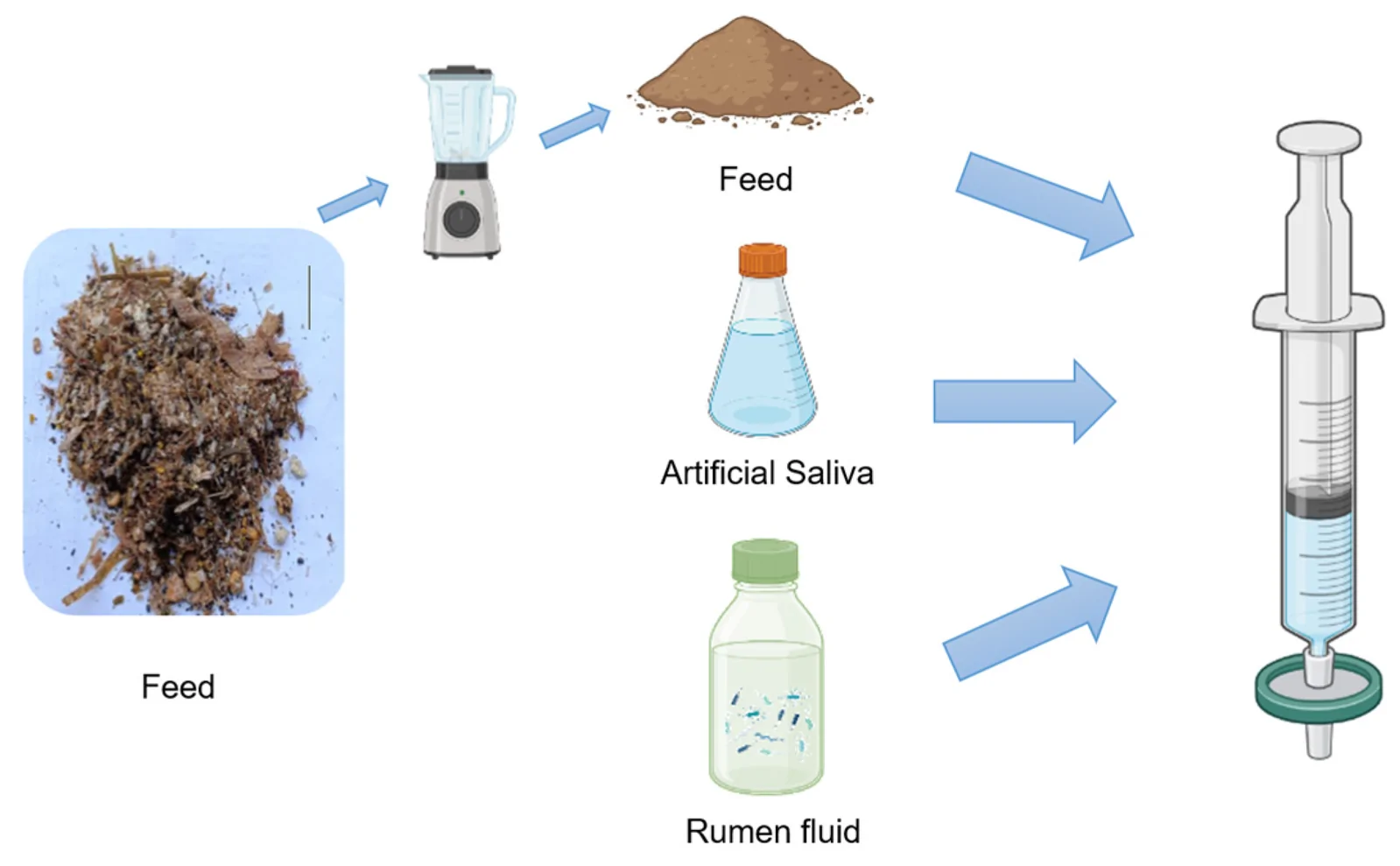
Representation of the quantification of total gas produced.

Gas volume was measured by reading the plunger displacement on the graduated 60 mL syringe (precision: ±0.5 mL) at each time point (3, 6, 9, and 12 h) following the methodology described by Theodorou et al. [21]. Briefly, 250 mg of the feed sample from each treatment diet (CON, 5% PSM and 10% PSM) was fermented in triplicate. An additional blank without a feed sample was included, bringing the total to 12 syringes with a 60 mL capacity. A mixture of 18.75 mL of artificial saliva [24] and 6.25 mL of ruminal fluid was carefully injected into each syringe (Figure 3). Subsequently, air bubbles were expelled, and the syringes were sealed [21].

pH was measured using a Mettler Toledo SevenCompact pH meter (model pH/ion S220; Mettler Toledo, Greifensee, Switzerland). The equipment was calibrated before use with standard buffer solutions (pH 1.68, 4.01, 7.00, and 10.01). For each measurement, the electrode was rinsed with distilled water before and after each determination, fully immersed in the sample, and the pH value was recorded after stabilisation (1–2 min). pH measurements were taken at 3, 6, 9, and 12 h of incubation. This incubation time was chosen to assess early-stage fermentation and gas production, focusing on the rapid initial phase of microbial activity.

### 2.5. Statistical Analysis and Visualisation

Data organisation and preliminary calculations were conducted using Microsoft Excel 2016 (Office 365, Version 16.0; Microsoft Corporation, Redmond, WA, USA). Performance variables, including individual body weights, daily group feed intake, and *in vitro* measurements such as gas volumes and pH, were compiled in this software. Descriptive statistics were calculated for key performance indicators: daily weight gain (ADG), Average daily feed intake (ADFI), and feed conversion ratio (FCR). The assumptions of normality (Shapiro–Wilk test) and homogeneity of variances (Levene’s test) were assessed. A one-way analysis of variance (ANOVA) was used to test the effect of treatment on ADG, *in vitro* gas production and overall pH. The statistical model included treatment as a fixed effect. Where assumptions were met, Tukey’s Honestly Significant Difference (HSD) test was used for pairwise comparisons. For gas production and pH, separate ANOVAs were conducted at each time point (3, 6, 9, and 12 h).

DeepSeek-V3.2 was used to generate Python code for statistical analyses, including power analysis, ANOVA, normality tests, and graphical outputs. Subsequent inferential statistical analysis and data visualisation were performed using Python (version 3.8.10; Python Software Foundation, Wilmington, DE, USA) within the Spyder (version 5.5.4) integrated development environment. The analysis used several scientific libraries: pandas for data manipulation, numpy for numerical operations, and scipy.stats for conducting Analysis of Variance (ANOVA). Visualisations were generated using matplotlib: pyplot, scipy.stats and statannotations.

All statistical analyses were independently verified using IBM SPSS Statistics (Version 21.0.0.0, 64-bit; IBM Corp., Armonk, NY, USA, 2012) to confirm the results obtained from the Python code. Differences among treatment means were evaluated using Tukey’s HSD post hoc test. For the *in vitro* assay, each treatment had three replicates (*n* = 3). For *in vivo* ADG, the individual steer was the experimental unit (*n* = 12). For all inferential tests, results with a *p*-value of less than 0.05 were considered statistically significant.

### 2.6. Feed Cost Calculations

Feeding cost as a percentage of the revenue generated from live weight gain was calculated for each dietary treatment (Section 3.6). This indicator was obtained by dividing the cost of the ration (USD per animal per day) by the value of the daily weight gain (USD per animal per day), which, in turn, was derived by multiplying the average daily gain by the market price per kilogram of live weight Equation (1). The resulting ratio was then multiplied by 100 to express the feeding cost as a percentage.

(1) $$\mathrm{Feeding}\;\mathrm{cost}\;(\%)=\left(\frac{C_{\mathrm{ration}}}{ADG\times P_{\mathrm{live}\;\mathrm{weight}}}\right)\times 100$$

where:

$C_{\mathrm{ration}}$ = cost of the ration (USD·animal^−1^·day^−1^).

ADG = average daily gain (kg·animal^−1^·day^−1^).

$P_{\mathrm{live}\;\mathrm{weight}}$ = market price per kg of live weight (USD·kg^−1^).

Average daily feed intake (ADFI) reported in Section 3.6 was calculated from pen-level fresh matter intake divided by the number of animals per group (*n* = 12), and then converted to a dry matter basis using the moisture content of each diet (Table 1). Average daily gain (ADG) was calculated for each individual steer (*n* = 12 per treatment) as the difference between final and initial body weight divided by 45 days (ADG = (BW_final − BW_initial)/days). Feed conversion ratio (FCR) was then calculated as the ratio of ADFI (kg DM animal^−1^ day^−1^) to ADG (kg animal^−1^ day^−1^) (Section 3.6).

## 3. Results and Discussion

Animals exhibited normal growth throughout the 45-day feeding trial, with body weight increasing from 416 ± 30 kg to 493 ± 31 kg. Furthermore, no clinical signs, behavioural alterations, or health issues were observed during the experimental period.

### 3.1. Feed Intake

**Figure 4:**
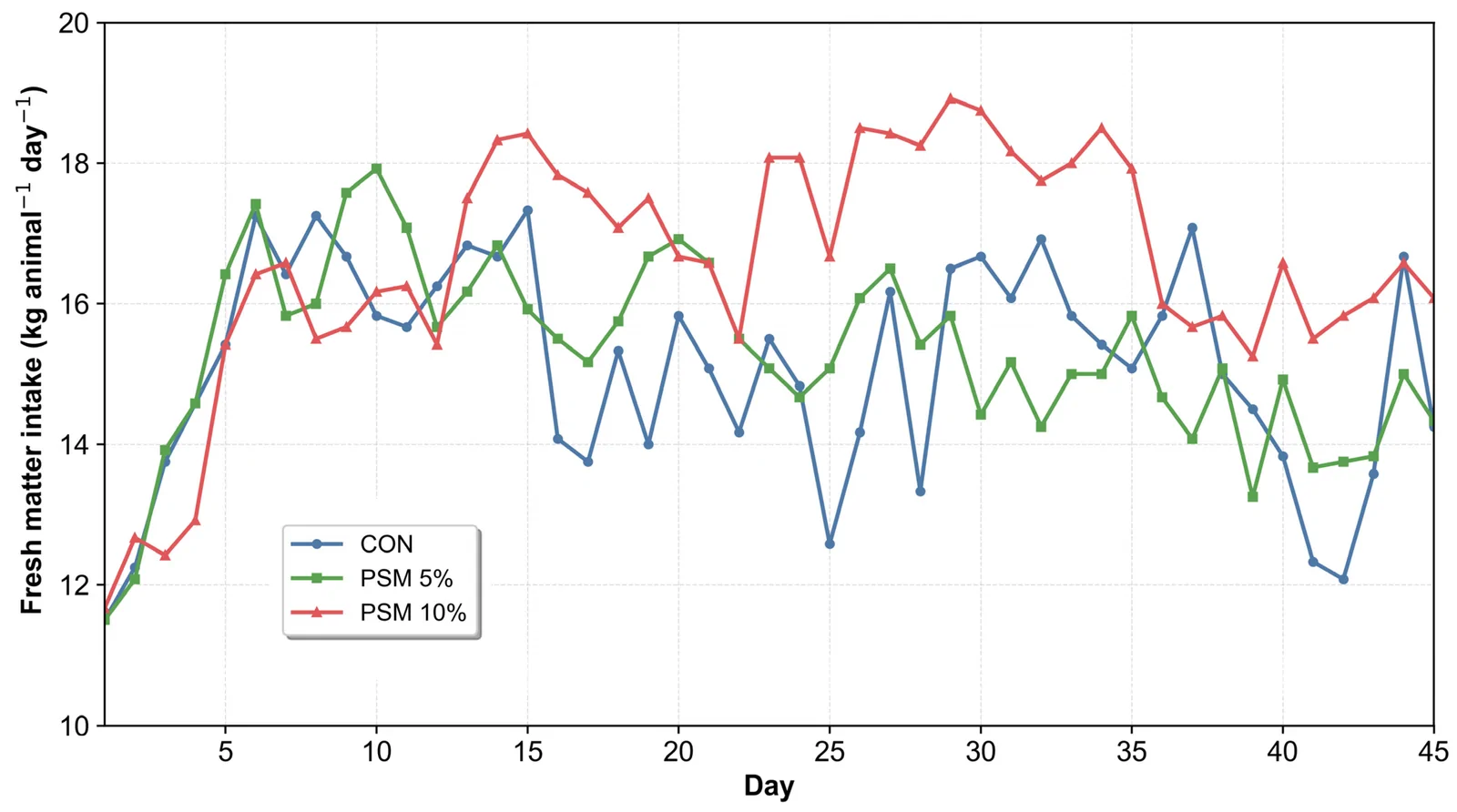
Average Daily Feed Intake (ADFI) of fresh matter (kg animal^−1^ day^−1^) during the experimental period (0–45). Values are group means based on daily pen feed consumption. CON: Control diet; PSM5%: yellow passion fruit seed meal at 5%; PSM10%: yellow passion fruit seed meal at 10%.

Fresh feed intake was measured at the group level (*n* = 1 per treatment) and therefore could not be statistically tested. From days 0 to 45, daily fresh feed consumption was recorded for each treatment group (Figure 4). The 10% PSM group had the highest mean overall fresh-matter intake, at 199 ± 21 kg, followed by the 5% PSM and CON groups, which consumed 15 and 17 kg less, respectively. Numerically, the 10% PSM group consumed 9,34% more fresh matter than the CON group. Although this numeric difference is present, the absence of individual intake data limited statistical inference. We hypothesise that this numerical difference may be attributable to the higher crude fat content of the 10% PSM diet (Table 1), a finer particle size, or improved palatability in the PSM diets. However, these variables were not measured in the present study, and further research is required to confirm these mechanisms.

The dietary moisture levels (Table 1) may influence intake patterns. In this case, the observed group dry matter intake, which increased with decreasing moisture content (CON: 99.32 kg, 5% PSM: 111.30 kg, 10% PSM: 122.21 kg), is consistent with the typical inverse relationship between moisture content and dry matter intake in ruminant diets [25]. This pattern suggests that the higher dry matter content of the PSM-based diets likely contributed to their increased voluntary intake. However, the extent of the rise in dry matter intake exceeds what would be expected from moisture differences alone, indicating that other factors, such as improved palatability or a more favourable fibre composition in the PSM, likely acted together to influence the observed differences in dry matter intake. Indeed, the PSM diets were observed to have a visibly finer particle size compared to the CON diet (Figure S3). While not quantitatively assessed in this experiment, a reduction in particle size is a well-established factor that promotes higher feed intake in ruminants [26]. Therefore, this physical characteristic may explain the increased consumption observed in the PSM treatments.

The consistently higher average intake for the 10% PSM treatment, especially during the second phase of the study, can be attributed to improved palatability and greater stability in ruminal fermentation. This effect is likely due to the nutritional profile of passion fruit seed meal, which is high in crude fibre (34.91%), ether extract (24.97%), and soluble carbohydrates [12]. We hypothesise that this particular combination of nutrients and bioactive compounds promotes a more stable rumen environment and an optimal passage rate, both of which are known to encourage voluntary intake in ruminants [27,28].

### 3.2. Average Daily Weight Gain

**Figure 5:**
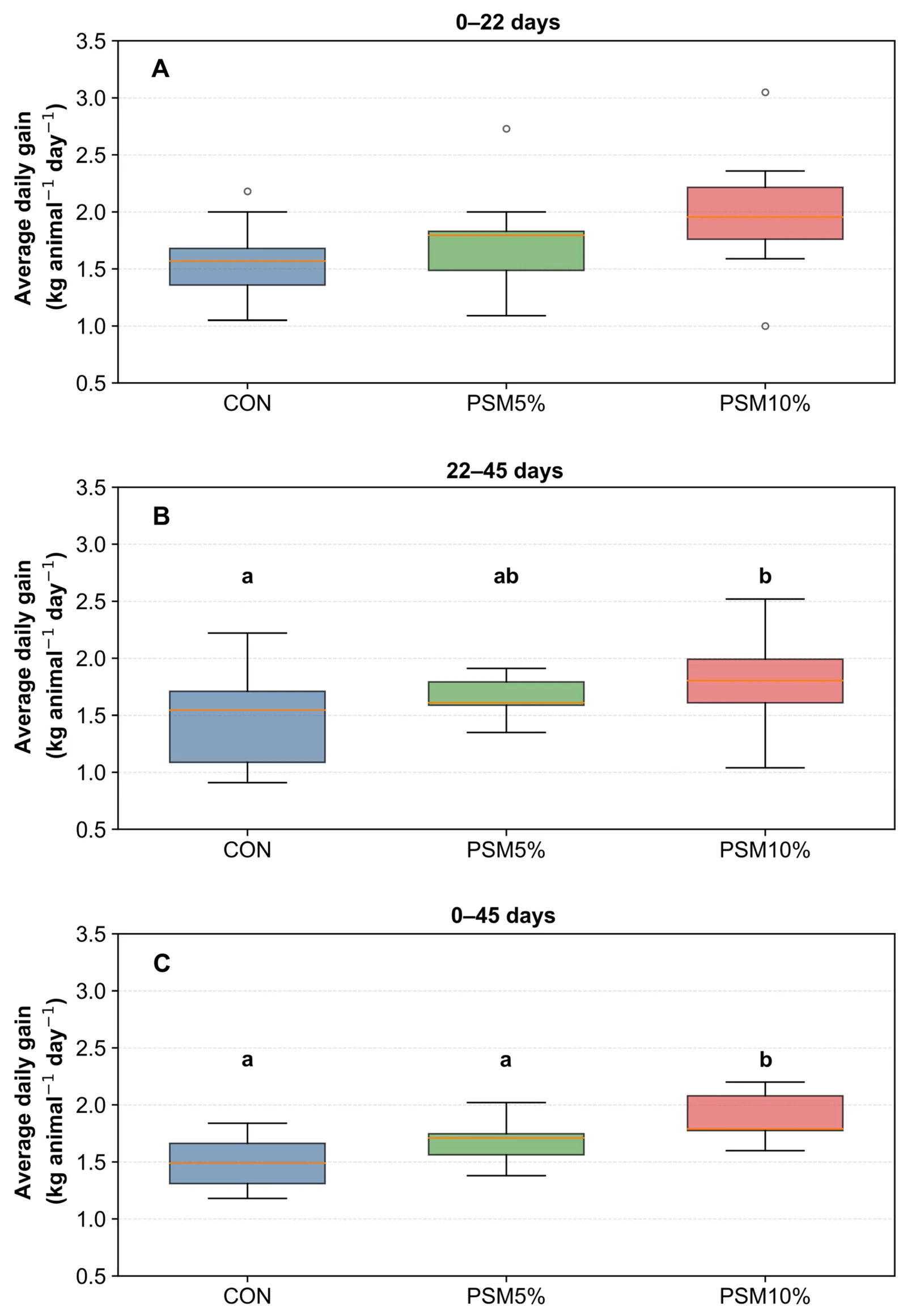
Average daily gain (ADG) kg animal^−1^ day^−1^ The averages were calculated based on the individual weights of the 12 animals per treatment group at 0–22 (**A**) and 22–45 days (**B**). The overall results 0–45 days are shown in (**C**). Boxes represent the interquartile range (25th–75th percentile), the horizontal orange line within each box indicates the median, and whiskers extend to 1.5× the interquartile range. Individual points beyond the whiskers are shown as outliers. Values with no common letters in each box are significantly different (*p* < 0.05). Panel (**A**) shows no significant differences among treatments (*p* = 0.061); therefore, no letters are displayed. Panels (**B**,**C**) show significant differences (*p* < 0.05), indicated by different letters. CON: Control diet; PSM5%: yellow passion fruit seed meal at 5%; PSM10%: yellow passion fruit seed meal at 10%.

Regarding the average daily gain (ADG), there was a significant effect of PSM in the second period (22–45 days) (*p* = 0.033) and the overall period (*p* = 0.00015) (Figure 5A). However, there was no statistically significant effect in the first period (*p* = 0.061). This might be attributed to the short adaptation period (10 days), which may have affected it and then became evident in the second period (Figure 5B). In agreement, Tayengwa et al. [29] reported that feeding dried grape pomace (150 g/kg DM) to Angus steers improved average daily gain (1.9 kg/day) compared to a control diet (1.5 kg/day), with dried citrus pulp producing similar results (1.8 kg/day). The improved growth performance was attributed to increased dry matter intake. These results align with our observation that including PSM at 10% significantly improved ADG (1.9 vs. 1.5 kg/day). On the other hand, Alves et al. [17] also reported improved ADG (1.30 kg/day) when fresh passion fruit peel and seeds replaced forage entirely in Nelore steers. However, the use of unprocessed by-product as a forage substitute rather than processed PSM as a partial concentrate replacement limits direct comparison. By contrast, Abreu Filho et al. [30] reported no effect on ADG (0.96–1.07 kg/day) with up to 30% palm kernel cake inclusion, suggesting that PSM may offer distinct nutritional properties compared to other agro-industrial by-products, possibly due to its soluble fibre and bioactive compounds (Figure 1; Table 1) [8]. These findings position PSM as a promising feed ingredient for improving growth performance in beef steers.

### 3.3. In Vitro Fermentation Effect on Gas and pH

**Figure 6:**
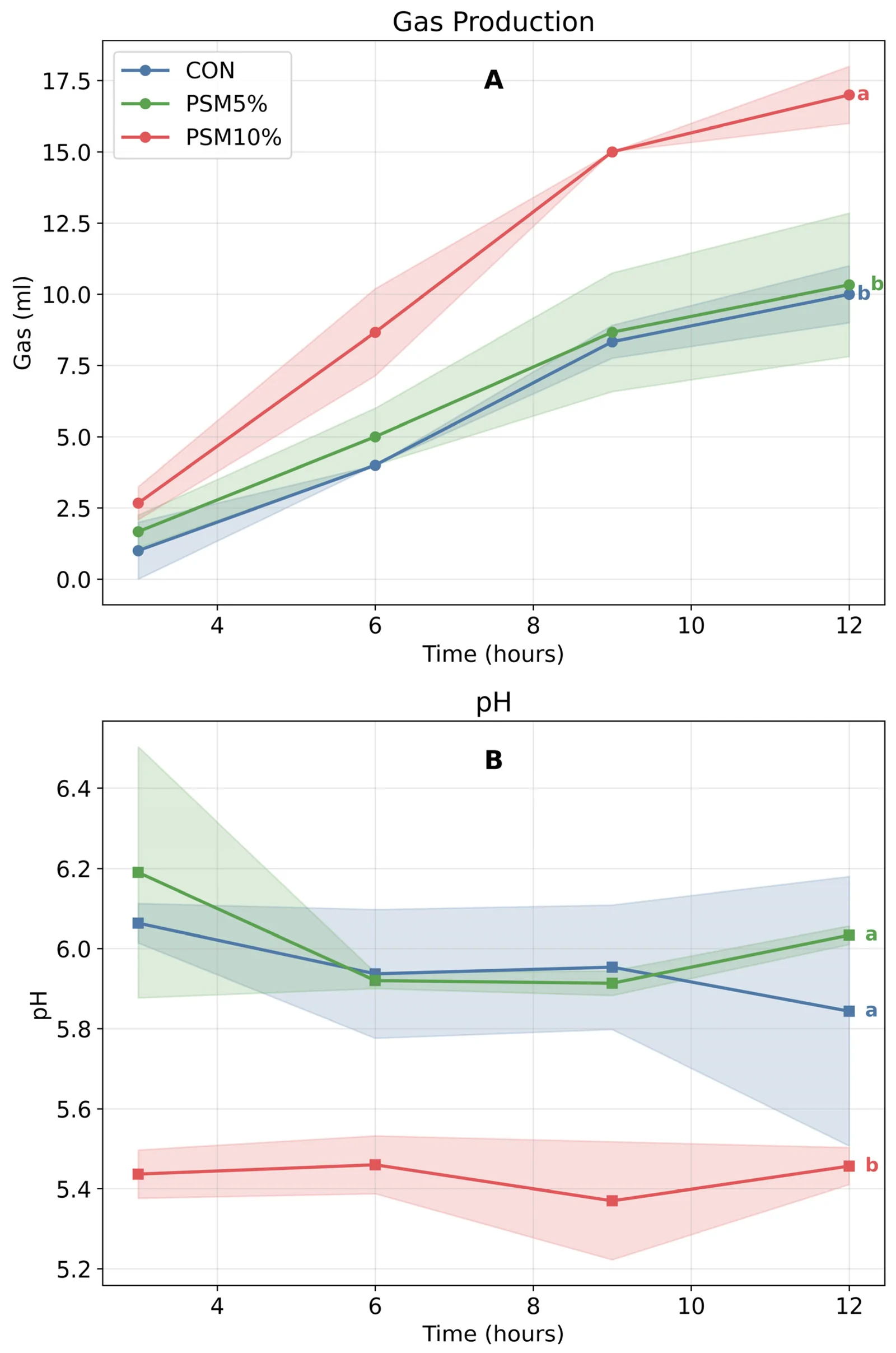
*In vitro* gas production (**A**) and pH (**B**) profiles. Values represent the mean of three replicates; shaded areas indicate the standard deviation of 3 replicates. CON: Control diet; PSM5%: diet with 5% yellow passion fruit seed meal; PSM10%: diet with 10% yellow passion fruit seed meal. For each panel, lines denoted by different letters are significantly different (*p* < 0.05). Statistical comparisons were based on (**A**) the cumulative gas volume at 12 h and (**B**) the overall mean pH across all time points (3, 6, 9, and 12 h).

At 12 h, cumulative gas production was 17.0 ± 1.00 mL for 10% PSM, 10.33 ± 2.52 mL for 5% PSM, and 10.00 ± 1.00 mL for CON (Table 2, Figure 6A). Pairwise comparisons showed that 10% PSM differed significantly from both CON (*p* = 0.02) and 5% PSM (*p* = 0.01), while CON and 5% PSM did not differ (*p* = 0.43).

The gas production volume exhibited a steady upward trend across all treatments during the 12 h experimental period. The 10% PSM treatment produced the highest cumulative gas volume, which was 64.56% greater than that of the 5% PSM treatment. Conversely, the gas volumes from the 5% PSM and CON treatments were remarkably similar, with only a marginal difference of 0.33 mL, though both showed considerable measurement variability (Table 2, Figure 6A). At the same time, a general decline in pH was noted, although this trend showed greater variability than the gas measurements (Table 3, Figure 6B). Additionally, considering the overall mean pH across the 3–12 h incubation period, the lowest value was observed in the 10% PSM treatment (5.43 ± 0.06), followed by CON (5.95 ± 0.13) and 5% PSM (6.01 ± 0.08) (Table 3).

The gas production and pH data reveal an effect of PSM inclusion on *in vitro* fermentation. Gas production and pH reduction are direct indicators of microbial fermentative activity. The significantly higher cumulative gas volume and correspondingly lower overall mean pH in the 10% PSM treatment indicate a substantially more intense and active fermentation process than in both the CON and 5% PSM treatments. The fact that CON and 5% PSM yielded statistically similar gas production and pH suggests that a 5% inclusion is insufficient to meaningfully alter microbial activity. In contrast, the 10% inclusion of PSM likely provided sufficient mass of fermentable substrates, surpassing a threshold that may have stimulated greater metabolic activity and increased gas production. Although the gas production and pH results indicate differences among treatments, these findings should be interpreted with caution given the limited number of replicates (*n* = 3 per treatment). The results are consistent with the hypothesis that 10% PSM is more fermentable, but further studies with larger replication are needed to confirm these findings.

We hypothesise that the lower average pH and higher total gas production in the 10% PSM diet could be linked to a higher dietary NDF content, as PSM has a high NDF level (Figure 1). This suggests that including PSM at 10% likely increased the diet’s overall NDF, providing more fermentable substrate for rumen microbiota and potentially boosting volatile fatty acid (VFA) and gas production (Table 2, Figure 6A). Although VFA levels were not directly measured, the reduced mean pH (Table 3, Figure 6B) aligns with increased fermentation activity. The VFA produced may also have contributed, at least in part, to the higher ADG observed throughout the study period (Figure 5C). Nonetheless, because NDF was measured only for the individual ingredient, not for the complete diets, this hypothesis warrants further investigation.

As this represents the first experimental approach using PSM as a feed ingredient, the 12 h sampling interval captures the initial phase of gas production but does not permit further observation of the latter effects of substrate [31,32]. Therefore, applying a mixed non-linear kinetic model to these data would be premature, as accurate parameter estimation requires a complete fermentation time course encompassing the lag, exponential, and plateau phases [33,34]. Therefore, our analysis focused on comparing cumulative gas volumes at 12 h and mean pH values, approaches valid for an initial study [32] and allow us to identify differences in curve shapes beyond the observed range [31].

### 3.4. In Vitro Fermentation Effect on the Bromatological Profile

The fermentation process altered the nutritional profiles of all experimental diets, characterised by reductions in crude protein, ether extract, and ash, alongside a significant increase in crude fibre (Table 4). These effects were expected, as in the closed system, with the exception of ash.

The post-fermentation reduction in crude protein indicates intense microbial activity, in which nitrogenous compounds were likely initially utilised for microbial protein synthesis and eventually lost as ammonia, thereby depleting crude protein content [35]. Concurrently, ether-extract fractions were almost entirely degraded (≈95% reduction), suggesting active lipolysis and lipid utilisation by the fermentative microbiota [36]. This decline reflects the hydrolytic action of microbial lipases, which cleave ester bonds in triglycerides, releasing free fatty acids and glycerol [37]. In anaerobic environments, these fatty acids undergo beta-oxidation, producing acetyl-CoA, which is subsequently redirected toward volatile fatty acid synthesis (e.g., acetate, butyrate), thereby driving microbial energy production (ATP) and growth [38,39].

In contrast to protein and fat, crude fibre concentration increased markedly (up to 399%). This reflects a relative concentration effect rather than fibre synthesis, while the soluble fermentable components (non-structural carbohydrates, proteins, fats) are metabolised, recalcitrant fractions, such as lignin, some cellulose, and hemicellulose, remain and constitute a larger proportion of the residual dry matter [35,40]. Ash content after fermentation was excluded from Table 4 because the observed values did not follow the expected mass-balance trend (i.e., ash should increase as organic matter decreases), and were therefore considered unreliable.

### 3.5. Bioactive Compounds in PSM

Yellow passion fruit seeds contain bioactive compounds such as tannins and flavonoids [8]. Although we did not measure these compounds in the diets, their presence may have contributed to the observed improvements in growth performance, possibly by modulating ruminal fermentation. In fact, polyphenols exert dose-dependent effects in growing ruminants: moderate concentrations (20–45 g/kg DM) improve protein utilisation by reducing ruminal degradation, whereas excessive levels (>50 g/kg DM) depress intake and digestibility [13,15,16]. Further chemical characterisation is required to confirm their specific effects in this context.

### 3.6. Economic Assessment of PSM Inclusion

The inclusion of PSM improved economic efficiency over the 45-day experiment. This gain was driven by superior average daily gain (ADG; kg animal^−1^ day^−1^) in PSM-fed steers, which reduced the contribution of feeding costs to total cost per kilogram of live weight gain (Table 5). Calculations integrated ration cost, ADG, and producer payment prices according to Juan Jiménez (president of the Asociación de Ganaderos de Pedro Vicente Maldonado, ASOGAN-PVM, Ecuador; personal communication, 2026).

The inclusion of 5 and 10% PSM reduced the contribution of feeding costs to 57% and 46%, respectively, compared to a conventional diet. This reduction is consistent with findings that agro-industrial by-products are generally less expensive than conventional feeds like corn and soy [41]. However, the high moisture content of many by-products, including PSM, can pose challenges for storage and transport, potentially increasing logistical costs [41]. To address this, ensiling has been proposed as a viable fermentation method to preserve these abundant and economical materials [42]. While these by-products are valuable, their inclusion level must be carefully managed. For example, when evaluating pumpkin by-products (*Cucurbita maxima* Duchesne) as a low-cost, highly metabolizable energy source, researchers concluded that it could effectively replace sorghum, but its inclusion in diets should not exceed 30% on a dry matter basis [43].

The economic benefits of this strategy are supported by broader research. Replacing traditional ingredients with agro-industrial by-products can lower feeding costs by 5% to 20% [44]. Furthermore, incorporating these non-conventional feed resources into animal diets helps mitigate competition between human and animal feed [45]. This approach also contributes to sustainability, as substituting conventional ingredients such as corn and soy reduces demand for associated resources, including land, water, fuel, and fertilisers [46]. These findings highlight the potential of this agro-industrial by-product to optimise marginal cost–benefit relationships in animal production.

### 3.7. Future Approaches

Given the positive effects in ADG observed with 10% PSM inclusion in steer diets, it is plausible that the PSM inclusion rate could be increased to 12–15% to further reduce feeding costs while potentially maintaining average daily gain (ADG). However, the presence of anti-nutritional factors such as tannins and insoluble fibre may limit nutrient digestibility at higher inclusion levels. Therefore, further research is required to confirm the optimal inclusion rate that maximises economic returns without compromising animal performance.

The use of crossbred steers in the present study introduced genetic variability that may have influenced individual responses to the dietary treatments. Homogenising the animals’ genetics by using a single breed or a defined genetic line would have improved the study design by reducing individual variation and increasing the statistical power to detect treatment effects. Despite this genetic heterogeneity, consistent and meaningful conclusions were drawn from the data, supporting the observed responses to PSM supplementation.

The *in vitro* fermentation data were obtained using rumen fluid from a single donor animal, thereby limiting the generalisability of the results, as the composition of the rumen microbiota can vary among individuals. Future research should include multiple donor animals to account for this variability and enhance the broader applicability of the findings.

A 12 h incubation period was selected to evaluate early-stage fermentation and gas production, focusing on the immediate microbial response to the substrates. Although this provided useful insights into initial fermentative activity, it does not allow observation of substrate exhaustion or the plateau phase. Therefore, future experiments should extend the incubation period to 48–72 h to capture the full fermentation profile and estimate reliable kinetic parameters. Although the gas production and pH results indicate differences among treatments, these findings should be interpreted with caution given the limited number of replicates (*n* = 3 per treatment). The results are consistent with the hypothesis that 10% PSM is more fermentable, but further studies with larger replication are needed to confirm these findings.

*Passiflora edulis* bioactive compounds, including passion fruit seed meal (PSM), may have exerted an effect in both *in vivo* and *in vitro* trials. Tannins and flavonoids were reported as the predominant compounds in Brazilian yellow passion fruit by-products [8]. As bioactive compounds were not analysed, chemical characterisation of Ecuadorian PSM would be required to identify those that might modulate ruminal biochemistry.

Future investigations should explore the effects of yellow PSM supplementation in dairy cattle, as their distinct metabolic and rumen fermentation profiles may yield different but equally valuable responses. Likewise, evaluating PSM across diverse genetic lines is a promising research direction for better understanding how genetic background influences the bioactivity of tannins and flavonoids. Such studies will help to maximise the applicability of PSM as a cost-effective and functional feed ingredient across the ruminant production sector.

## 4. Conclusions

The use of fruit by-products, such as yellow passion fruit seed meal (PSM), in ruminant diets presents a dual opportunity as it reduces feed costs while alleviating competition between human and livestock feed sources. In the present study, dietary inclusion of up to 10% PSM was associated with numerically higher group-level feed intake and significantly higher average daily gain, with the highest inclusion level showing the most pronounced effects in fattening steers. These improvements were observed alongside a substantial reduction in feeding costs, demonstrating that PSM can enhance productive efficiency without compromising economic viability.

The *in vitro* gas production results, under the conditions tested, indicated that PSM is fermentable, and the *in vivo* performance improvements support its potential as a feed ingredient. However, caution should be exercised when interpreting the *in vitro* results in light of the limitations discussed in Section 3. 

Taken together, the findings position PSM as a sustainable and cost-effective feed ingredient for local beef cattle systems. Future research should explore the long-term effects of this locally sourced by-product across different production systems and genetic backgrounds.

## Figures and Tables

**Table 1:** Composition of the experimental diets with yellow passion fruit seed meal (PSM).

Ingredients		Diets	
	CON (%)	5% PSM	10% PSM
Yellow passion fruit seed meal (PSM)	-	5.00	10.00
Corn silage	40.00	30.00	35.00
Corn grits	22.00	34.60	29.00
Rice bran	5.00	10.00	10.00
Cocoa husk	13.00	10.00	10.00
Soybean pellet	8.00	5.00	-
Corn	8.00	-	-
Molasses	1.00	1.00	3.00
Palm oil	2.00	3.00	1.00
Urea	-	0.40	1.00
Mineral Mix ^1^	1.00	1.00	1.00
**Nutritional composition (g/kg DM)—Calculated**			
Crude Protein	122	121	123
Crude Fiber	172	188	204
Crude Fat	62	87	79
Ash	54	54	54
Metabolizable Energy ^2^	2.7	2.7	2.7
**Nutritional composition (g/kg DM)—Analyzed ^3^**			
Moisture ^4^	45.43%	39.51%	38.59%
Crude Protein	165	149	159
Crude Fiber	130	112	128
Ether Extract	93	92	102
Ash	49	45	47
NFE ^5^	564	603	564

^1^ Mineral Mix composition (per kg of mix): Ca: 20 g; Cl: 17 g; P: 2 g; Mg: 0.7 g; Na: 11 g; Minerals: 2 g; Flavouring: 0.001. ^2^ Metabolizable energy expressed in Mcal/kg of dry matter (DM). ^3^ Analysed compositions from Agrolab Laboratory. ^4^ Moisture content was determined on the original sample and is expressed as a % of fresh weight. ^5^ NFE: Nitrogen Free Extract.

**Table 2:** Cumulative gas production (mL, mean ± SD) over time and cumulative volume at 12 h.

Treatment	Gas 3 h	Gas 6 h	Gas 9 h	Gas 12 h (Cumulative) ^1^
CON	1.00 ± 1.00	4.00 ± 0.00	8.33 ± 0.58	10.00 ± 1.00
PSM5%	1.67 ± 0.58	5.00 ± 1.00	8.67 ± 2.08	10.33 ± 2.52
PSM10%	2.67 ± 0.58	8.67 ± 1.53	15.00 ± 0.00	17.00 ± 1.00
*p*-value (ANOVA) ^2^	0.083	0.004	<0.001	0.003

^1^ The cumulative volume at 12 h was used for statistical comparisons among treatments (letters in Figure 6A). ^2^ One-way ANOVA comparing the three treatments at each time point (*n* = 3 replicates per treatment). Significant differences were considered at *p* < 0.05.

**Table 3:** pH values (mean ± SD) over time, overall mean, and *p*-values for treatment effect at each time point.

Treatment	pH 3 h	pH 6 h	pH 9 h	pH 12 h	Overall Mean (3–12 h) ^1^
CON	6.06 ± 0.05	5.94 ± 0.15	5.95 ± 0.15	5.84 ± 0.32	5.95 ± 0.13
PSM5%	6.19 ± 0.30	5.92 ± 0.02	5.91 ± 0.03	6.03 ± 0.02	6.01 ± 0.08
PSM10%	5.44 ± 0.04	5.46 ± 0.07	5.37 ± 0.15	5.46 ± 0.04	5.43 ± 0.06
*p*-value (ANOVA) ^2^	0.005	0.002	0.002	0.030	0.001

^1^ The overall mean corresponds to the average of the three replicates across the four measurement times (3, 6, 9, and 12 h). This value was used for statistical comparisons using ANOVA and Tukey’s HSD post hoc test (letters in Figure 6B). ^2^ One-way ANOVA comparing the three treatments at each time point (*n* = 3 replicates per treatment). Significant differences were considered at *p* < 0.05.

**Table 4:** *In vitro* fermentation effect on the bromatological values. ‘After’ values were determined on the solid residue after fermentation, which was filtered and dried before analysis.

Treatment	Parameter	Before	After	Absolute Change (±)	%
CON	Crude Protein	165	14	−151	−92%
	Crude Fiber	130	575	445	342%
	Ether Extract	93	5	−88	−95%
PSM5%	Crude Protein	149	14	−135	−91%
	Crude Fiber	112	559	447	399%
	Ether Extract	92	4	−88	−96%
PSM10%	Crude Protein	159	12	−147	−92%
	Crude Fiber	128	569	441	345%
	Ether Extract	102	4	−98	−96%

CON: Control diet; PSM5%: diet with 5% yellow passion fruit seed meal; PSM10%: diet with 10% yellow passion fruit seed meal.

**Table 5:** Effect of treatments on feeding costs and payment per kilogram of live weight.

Treatment	Ration Cost (USD)	ADFI (kg DM Animal^−1^ day^−1^) ^1^	ADG (kg Animal^−1^ day^−1^) ^2^	FCR (kg DM/kg ADG) ^3^	USD per kg Live Weight	Value of Gain (USD)	Feeding Cost (% of Revenue)
CON	3.00	8.28	1.5	5.52	2.42	3.63	83
PSM5%	2.35	9.28	1.7	5.46	2.42	4.11	57
PSM10%	2.10	10.18	1.9	5.36	2.42	4.60	46

CON: Control diet; PSM5%: diet with 5% yellow passion fruit seed meal; PSM10%: diet with 10% yellow passion fruit seed meal. DM: Dry matter; USD: United States Dollar. ^1^ ADFI: Average daily feed intake (ADFI) was calculated as group-level fresh matter intake divided by the number of animals per group (*n* = 12), and then converted to a dry matter basis using the moisture content of each diet (Table 1). ^2^ ADG: Average daily weight gain was calculated for each steer (*n* = 12 per treatment) as (final BW − initial BW)/45 days. ^3^ FCR: Feed conversion ratio = ADFI (kg DM animal^−1^ day^−1^)/ADG (kg animal^−1^ day^−1^). Lower values indicate better feed efficiency.

## Data Availability

The raw data supporting the conclusions of this article will be made available by the authors on request.

## Supplementary Materials

The following supporting information can be downloaded at: https://www.mdpi.com/article/10.3390/ani16162513/s1.

## Abbreviations

The following abbreviations are used in this manuscript:

ADG	Average Daily Gain (kg animal^−1^ day^−1^)
ADFI	Average Daily Feed Intake (kg animal^−1^ day^−1^)
ADF	Acid Detergent Fibre
AOAC	Association of Official Analytical Chemists
ASOGAN-PVM	Livestock Breeders Association of Pedro Vicente Maldonado
BW	Body Weight
CP	Crude Protein
DM	Dry Matter
EE	Ether Extract
FCR	Feed conversion ratio
NDF	Neutral Detergent Fibre
NFE	Nitrogen Free Extract
PSM	Yellow Passion Fruit Seed Meal (*Passiflora edulis* f. *flavicarpa* by-product)
VFA	Volatile Fatty Acids
CON	Control Diet (0% PSM inclusion)
PSM5%	Diet with 5% Yellow Passion Fruit Seed Meal
PSM10%	Diet with 10% Yellow Passion Fruit Seed Meal
