# Supplementary material for: Yellow Passion Fruit Seed Meal (Passiflora edulis f. flavicarpa) as a Feed Ingredient for Fattening Steers: Effects on Intake, Weight Gain, In Vitro Fermentation and Feed Cost
Source: Animals (Basel). 2026 Aug 12;16(16):2513. doi: 10.3390/ani16162513 (PMC13508961; doi:10.3390/ani16162513)
Supplement: Supplementary file 1 [file animals-16-02513-s001.zip › Figure S1_ Seidlaboratory .pdf]

SEIDLABORATORY CÍA. LTDA.

SERVICIO INTEGRAL DE LABORATORIO

# INFORME DE ENSAYO NR.249836

| INFORMACIÓN PROPORCIONADA POR EL CLIENTE | | | |
|---|---|---|---|
| **Cliente:** | DURAN SALAZAR VICTOR HUGO | | |
| **Dirección:** | SANTO DOMINGO DE LOS TSACHILAS / SANTO DOMINGO / RIO TOACHI / SAQUISILI 178 Y ATUNTAQUI | | |
| **Nombre Producto :** | PASTA INTEGRAL DE MARACUYÁ - 08 DE MARZO 2022 | | |
| **Fecha de Elaboración:** | ND | **Fecha de Caducidad:** | ND |
| **Lote:** | ND | **Contenido Declarado:** | ND |
| **Material Envase:** | FUNDA ZIPLOC CERRADA | **Forma de Conservación:** | Ambiente |

| INFORMACIÓN DE LA MUESTRA | | | |
|---|---|---|---|
| **Código Laboratorio :** | 249836-1 | **Contenido Encontrado:** | 302.1 Gramos |
| **Fecha Recepción:** | 2022/04/12 | **Fecha Inicio Ensayo:** | 2022/04/12 |
| **Condiciones Ambientales de llegada de la muestra:** | 20 ºC | **Muestreo:** | Es responsabilidad del cliente y, los resultados aplican a la muestra entregada por el cliente tal como se recibió |

| ENSAYOS FFQQ | MÉTODO | ACREDITACIONES | | UNIDAD | RESULTADO |
|---|---|---|---|---|---|
| | | A2LA | SAE | | |
| CARBOHIDRATOS | CALCULO | * | * | % | 50.00 |
| CENIZA | SE.MI | * | * | % | 1.49 |
| ENERGIA TOTAL | CALCULO | * | * | Kcal/100g | 493 |
| FIBRA CRUDA | SE.MI (AOAC 978.10) | * | * | % | 34.91 |
| GRASA TOTAL | SE.MI | * | * | % | 24.97 |
| HUMEDAD | SE.MI | * | * | % | 6.56 |
| PROTEINA F=6.25 | SE.MI | * | * | % | 16.98 |

**INCERTIDUMBRE**

| PARAMETRO | INCERTIDUMBRE | |
|---|---|---|
| CENIZA | L+- 4.0% (Rangos Mayores al 5.0% ) | La incertidumbre expandida reportada esta basada en una incertidumbre tipica multipicada por un factor de cobertura K=2, proporcionando un nivel de confianza de un 95%. |
| | L+- 7.0% (Rangos Menores o igual al 5.0% ) | |
| GRASA TOTAL | L+- 11.06 (Rangos Menores al 5.0% ) | |
| | L+- 5.61 (Rangos Mayores al 5.0% ) | |
| HUMEDAD | L+- 5.0% (Rangos Mayores al 5.0% ) | |
| | L+- 8.0% (Rangos Menores al 5.0% ) | |

NS: No solicita el cliente/ ND: No declara.

"Los ensayos marcados con (*) NO están incluidos en el alcance de la acreditación"

Datos tomados de F-RG-01 pág. 157/ C-RG-03 pág. 326 / H-RG-02 pág. 673 / PDU-RG-01 pág. 439 / GE-RG-03 pág. 322

Los resultados expresados arriba tienen validez solo para la muestra analizada en condiciones específicas no siendo extensivo a cualquier lote

El laboratorio no se responsabiliza por la representabilidad de la muestra respecto a su origen y sitio del cual fue tomado

Este informe no será reproducido, excepto en su totalidad con la aprobación del Director Técnico

"SEIDLABORATORY CIA LTDA no se responsabiliza por la información declarada por el cliente"

- Tiempo de almacenamiento de informes: Cinco años a partir de la fecha de ingreso de la muestra

22/04/22

**FECHA EMISIÓN**

Atentamente.

Firmado digitalmente por:ANA GABRIELA VALENCIA MURGUEYTIO
Fecha y hora: 2022-04-22 17:49:33

*Muestra 249836-1 de 249836-1*



*Confidencialidad e Imparcialidad*
*Seidlaboratory Cia. Ltda. asume la responsabilidad legal sobre la gestión de la información obtenida o creada durante la realización de actividades del laboratorio a partir de la(s) muestra(s) ensayada(s), información considerada como confidencial y de propiedad del cliente. Seidlaboratory Cia. Ltda. se compromete a usar dicha información únicamente de la manera y para los propósitos acordados por las partes; en caso de controversias, las partes se someterán al Centro de Mediación de la Cámara de Comercio de Quito.*
*Tiempo de permanencia de las muestras en el laboratorio*
*Muestras perecibles: 8 días calendario; Muestras no perecibles: 30 días calendario. Si desea repetición de algún parámetro, se debe generar una solicitud en el periodo estipulado.*
*Para consultas, quejas o sugerencias, favor comunicarse a los siguientes correos:*
*Dirección de Calidad directordecalidad@seidlaboratory.com.ec; Gerencia General gerenciageneral@seidlaboratory.com.ec; Servicio al Cliente servicioalcliente@seidlaboratory.com.ec*
*Melchor Toaza N61-63 entre Av. del Maestro y Nazareth      022476314 - 022483145 - 0995450911 - 0992750633*
